# Prone mechanical ventilation in acute brain injury

**DOI:** 10.1186/s13054-021-03530-8

**Published:** 2021-03-10

**Authors:** Varun Suresh, Saurabh Sharma

**Affiliations:** 1 Department of Anaesthesiology, Government Medical College, Thiruvananthapuram, Kerala 695011 India; 2 Department of Neuro-Anesthesia and Neuro-Critical Care, Kalinga Institute of Medical Sciences, Bhubaneswar, Odisha 751024 India

## Dear Editor,

We read with great interest the research work by Bernon and colleagues [1] that retrospectively analyses the influence of prone positioning (PP) on intracranial pressure (ICP) in acute brain injury. The study elucidates acute ICP changes within an interval of 1-h, following PP, as a safe limit to decide on pursuing this manoeuvre further in acute brain injury; nevertheless, we have a few concerns regarding this retrospective analysis.

We could not infer the exact technique of measurement of ICP in this retrospective study. Multiple invasive and non-invasive methods are available to measure ICP, each with its own strengths and limitations [2]. It is understood from the study results that seven patients in each group (raised ICP versus normal ICP) had external ventricular drains (EVD). Among patients with EVD, ICP is frequently measured using the same ventricular access [3]. Hence, we infer that to ensure uniformity of ICP data studied the authors must have used intra-parenchymal catheter-based technique of ICP measurement. We would also like to know whether such technique used was similar across all the study patients.

We also categorically comment on the wide heterogeneity among this small study population. The ICP dynamics and cerebral autoregulation vary widely across patients with traumatic brain injury, aneurysmal subarachnoid haemorrhage and hemorrhagic stroke. Hence, we presume this heterogeneity contributed to the insignificant results of PP on ICP among patients whom underwent major ICP lowering interventions like craniectomy (*p* = 0.595), barbiturate coma (*p* = 0.694) and osmotherapy (*p* = 0.440).

We would also argue against using a uniform positive end expiratory pressure (PEEP  = 10 mmHg) among patients with and without raised ICP. A high PEEP can contribute to disputed ICP readings in patients with autoregulatory failure as seen in some cases of intracranial hypertension [4, 5].

That the narration by Bernon and colleagues favour non-invasive methods of cerebral compliance assessment like the transcranial Doppler in acute brain injury, prior to protective lung ventilation interventions, is highly appreciated. Yet a clarification on our concerns shall add more to the understanding of the readers.

## Data Availability

Not applicable.
